# 质子射线与光子射线治疗肺癌的剂量学比较：*meta*分析

**DOI:** 10.3779/j.issn.1009-3419.2013.05.07

**Published:** 2013-05-20

**Authors:** 广伟 田, 楠 李, 光 李

**Affiliations:** 110001 沈阳，中国医科大学附属第一医院放疗科 Department of Radiation Oncology, First Affiliated Hospital of China Medical University, Shenyang 110001, China

**Keywords:** 质子, 光子, *meta*分析, 肺肿瘤, 放射治疗, Proton, Photon, *Meta*-analysis, Lung neoplasms, Radiotherapy

## Abstract

**背景与目的:**

由于缺乏质子治疗与传统光子治疗比较的临床随机对照研究，导致质子治疗在肺癌放疗中的应用没有充足的临床证据。本研究旨在探讨质子射线在肺癌放疗中的剂量学优势，以期为临床提供有价值的循证医学依据。

**方法:**

计算机检索Cochrane Library、PubMed、EMbase、中国生物医学文献数据库、中国学术期刊全文数据库和中国科技期刊数据库，同时辅助其它检索，搜集所有比较质子射线与光子射线治疗肺癌的剂量学研究的文章，应用RevMan 5.2软件对满足条件的数据进行*meta*分析。

**结果:**

6篇文献纳入本次研究。质子射线与光子射线三维适形放疗（three-dimensional conformal radiotherapy, 3D-CRT）技术比较，降低了双肺D_mean_（*MD*=-4.15, 95%CI: -5.56--2.74, *P* < 0.001）及V20、V10、V5（*MD*=-10.92, 95%CI: -13.23--8.62, *P* < 0.001）；降低了食管D_mean_及心脏D_mean_，差异有统计学意义。质子射线与光子射线调强放疗（intensity-modulated radiotherapy, IMRT）技术比较，V20、V10、V5在质子射线治疗组明显降低（*MD*=-3.70, 95%CI: -5.31--2.10, *P* < 0.001; *MD*=-8.86, 95%CI: -10.74--6.98, *P* < 0.001; *MD*=-20.13, 95%CI: -27.11--13.14, *P* < 0.001）；而相比于光子射线（IMRT），质子射线治疗肺癌在食管D_mean_差异无统计学意义，但心脏D_mean_明显降低，差异有统计学意义。

**结论:**

与目前普遍采用的光子射线（3D-CRT技术及IMRT技术）放疗相比，质子射线在治疗肺癌的剂量学方面有明显的优势，具有临床应用价值。

## Abstract (en)

**Background and objective:**

The clinical evidences are not sufficient on the proton beam therapy of lung cancer for lacking of the RCTs on the comparing the proton with the photon beam in lung cancer radiotherapy. The aim of this study is to evaluate the dosimetry superiority of the proton beam and provide more valuable evidences to the clinical researches.

**Methods:**

Clinical trails of dosimetric comparing between protons beam and photons beam for lung cancer radiotherapy were obtained from the Cochrane library, Pubmed, EMbase, CBM, CNKI, VIP, and Wan Fang databases. The data included in the study were evaluated and analyzed using the Cochrane Collaboration's RevMan 5.2 software.

**Results:**

Six trails were included. Compared to photon therapy (three-dimensional conformal photon radiotherapy, 3D-CRT), the proton therapy had a significantly lower total lung D_mean_ (*MD*=-4.15, 95%CI: -5.56--2.74, *P* < 0.001) and V20, V10, V5 (*MD*=-10.92, 95%CI: -13.23--8.62, *P* < 0.001); The V20, V10, V5 significantly decreased in proton therapy group. Compared to photon therapy (intensity-modulated photon radiotherapy, IMRT), V20, V10, V5 were also significantly lowered in proton therapy group (*MD*=-3.70, 95%CI: -5.31--2.10, *P* < 0.001; *MD*=-8.86, 95%CI: -10.74--6.98, *P* < 0.001; *MD*=-20.13, 95%CI: -27.11--13.14, *P* < 0.001); The esophagus D_mean_ was not lowered, while the heart D_mean_ decreased in proton therapy group.

**Conclusion:**

Comparing to photon beam radiotherapy (3D-CRT and IMRT), proton beam therapy is advantageous in dosimetry of the lung cancer radiotherapy and recommended for clinical applying.

与光子射线相比，质子射线具有Bragg峰的物理学特质，这种Bragg峰的优良剂量分布促使质子束的能量能集中在癌细胞处释放。质子治疗时将峰值部分对准肿瘤病灶处，肿瘤处受到最大的照射剂量，肿瘤前的正常细胞只受到1/3到1/2的峰值剂量，肿瘤后部的正常细胞基本上不受到任何伤害。这一特点使质子射线具备在放射治疗中提高肿瘤的照射剂量，减小正常组织受量的剂量学优势。从1990年第一台质子治疗机开始应用于临床^[1]^到目前为止，全球已有29个质子中心（我国拥有1个）^[2]^，预计到2014年，世界范围内将成立54个质子治疗中心^[3]^。目前质子治疗开始应用于中枢神经系统肿瘤、眼葡萄膜黑色素瘤、前列腺癌、非小细胞肺癌、胃肠道肿瘤、头颈部肿瘤、儿童肿瘤等的放疗。随着研究者们对质子治疗的优化，尤其是整合了4D-CT技术和图像引导放射治疗（image guide radiation therapy, IGRT）技术后，一些研究机构进行了质子治疗应用于肺癌放疗的临床研究，有文章对质子治疗肺癌进行系统分析^[4, 5]^，但是由于缺乏与传统光子治疗比较的临床随机对照研究，对质子治疗肺癌的临床优势很难下结论，2012年ASTRO会议关于质子治疗（protons beam therapy, PBT）的结论为：目前尚无充足的临床证据推荐质子治疗在肺癌中应用^[6]^。已有一些临床病例对照研究进行了质子射线与光子射线治疗肺癌的剂量学比较，为质子治疗在肺癌中的临床应用提供了理论基础。为此，本研究针对两种射线在治疗肺癌剂量学上的差异进行*meta*分析，以期为临床提供有价值的循证医学依据。

## 1 资料与方法

### 1.1 纳入与排除标准

#### 1.1.1 研究类型

已发表的比较质子射线与光子射线在治疗肺癌剂量学上的差异的文献。

#### 1.1.2 纳入标准

质子射线与光子射线治疗肺癌的临床研究，语种不限。原始文献中有明确的质子射线与光子射线（3D-CRT和IMRT）治疗肺癌的相关剂量学数据。

#### 1.1.3 排除标准

系统分析及综述，文献中无详细的相关剂量学数据。

### 1.2 观察指标

双侧肺总D_mean_、患侧肺D_mean_、V20、V10、V5、食管D_mean_及心脏D_mean_。

### 1.3 检索方法

以“lung neoplasms、lung carcinoma、lung cancer、proton”为检索词检索Cochrane图书馆（2012年第12期）、PubMed（1978-2012）、EMbase（1974-2012）。以“肺癌、肺肿瘤、质子”为检索词检索中国期刊全文数据库（1979-2012）、中国生物医学文献数据库（1979-2012）、中文科技期刊全文数据库（1989-2012）。

### 1.4 资料提取

由2位研究者独立对检索到的文献按照纳入与排除标准进行资料选择、数据提取、质量评价，如遇分歧通过讨论解决，必要时由第3位研究人员参与解决。

### 1.5 统计学方法

采用Cochrane协作网提供的软件包（RevMan 5.2）进行*meta*分析，将资料按照不同剂量学指标进行亚组分析。纳入研究结果之间的异质性采用χ^2^检验，*P*≥0.1为研究间具同质性，采用固定效应模型描述；*P* < 0.1为研究间具异质性，采用随机效应模型描述。

## 2 结果

### 2.1 文献检索及纳入文献的特征

初次检索获得351篇相关文献，阅读文献标题和摘要后初步纳入质子文章24篇，进一步阅读全文后排除文献18篇（无光子射线对照组3篇，综述9篇，无法提取数据6篇）。最终纳入6篇符合要求的文献。各纳入研究的特征见表 1。

**1:** 纳入研究的基本特征 The general characteristics of included studies

Included studies	Nation (study type)	Cases	Clinicopathological staging	Radiotherapy	Pathological types
Jiang 2006^[7]^	China (clinical control study)	5	NR	60 Gy/60 CGE	AD: 1 SCC: 3 Unknown: 1
Chang 2006^[8]^	America (clinical control study)	25	Stage Ⅰ：10 Stage Ⅲ：15	Phase Ⅰ: 66 Gy, 87.5 CGE Phase Ⅲ: 63 Gy, 74 CGE	NR
Wang 2009^[9]^	Japan (clinical control study)	24	Stage Ⅰ Ⅰa: 16 Ⅰb: 16	66 Gy/60 CGE	AD: 16 SCC: 6 Others: 2
Georg 2008^[10]^	Australia (clinical control study)	12	NR	45 Gy/3 f	NR
Register 2011^[11]^	America (clinical control study)	15	Stage Ⅰ：15	50 Gy/4 f	NSCLC NOS: 15
Roelofs 2012^[12]^	America, Norway, Canada (multicenter clinical control study)	25	Stage Ⅰ：4 Stage Ⅱ：3 Stage Ⅲ：18	70 Gy/70 CGE	AD: 5 SCC: 7 LC: 11 NSCLC NOS: 2
SCC: squamous cell carcinoma; AD: adenocarcinoma; LC: large cell carcinoma; NSCLC NOS: non-small cell lung cancer not otherwise specified; CGE: cobalt Gray equivalent; NR: not reported.					

### 2.2 *Meta*分析结果

#### 2.2.1 CTV及PTV的剂量分布比较

研究中关于质子射线与光子射线治疗肺癌CTV及PTV的剂量分布评价指标不同，由于数据有限，无法进行*meta*分析，本文仅做描述性分析，详细数据见表 2。

**2:** CTV及PTV的剂量分布比较 Dosimetric comparison of CTV and PTV

Studies	Statistical index	Data	*P*
Jiang 2006^[7]^	CI IC	CI: 3D-CRT *vs* IMRT *vs* PBT=1.54 *vs* 1.27 *vs* 1.21 IC: 3D-CRT *vs* IMRT *vs* PBT=0.4 *vs* 0.29 *vs* 0.59	NC
Wang 2009^[9]^	CTV covered by 90% isodose line (%) CTV covered by 95% isodose line (%)	3D-CRT *vs* PBT=99.0±0.4 *vs* 99.0±1.3 3D-CRT *vs* PBT=43.2±33 *vs* 86.4±12.5	0.57 < 0.001
Georg 2008^[10]^	CI	DIBH: 3D-CRT *vs* PBT=0.78±0.02 *vs* 0.53±0.08 SB+AC: 3D-CRT *vs* PBT=0.77±0.03 *vs* 0.54±0.08	< 0.001 < 0.001
Roelof 2012^[12]^	CN	3D-CRT *vs* PBT=0.25±0.07 *vs* 0.25±0.08 IMRT *vs* PBT=0.38±0.1 *vs* 0.25±0.08	> 0.999 < 0.001
CI: conformity index; IC: inhomogeneity coefficient; CN: conformation number; DIBH: deep inspiration breath hold; SB+AC: shallow breathing with abdominal compression; 3D-CRT: three-dimensional conformal photon radiotherapy; IMRT: intensity-modulated photon radiotherapy; PBT: proton beam therapy; NC: not calculable.			

#### 2.2.2 危及器官（肺）的剂量分布比较

3篇文章提供质子*vs* 光子（3D-CRT）治疗肺癌的双侧肺D_mean_、患侧肺D_mean_数据，异质性分析*P*值均 < 0.001，均采用随机效应模型分析，结果显示，双侧肺D_mean_比较：*MD*=-4.15，95%CI: -5.56--2.74，*P* < 0.001，质子明显优于光子（3D-CRT），差异有统计学意义；患侧肺D_mean_比较：*MD*=-1.10，95%CI: -2.40-0.20，*P*=0.10，差异无统计学意义；5篇文章提供质子*vs*光子（3D-CRT）治疗肺癌的V20、V10和V5数据，采用随机效应模式通过*meta*分析综合比较，其结果为*MD*=-10.92，95%CI: -13.23--8.62，*P* < 0.001，质子明显优于光子（3D-CRT），差异有统计学意义（图 1和图 2）。3篇文章提供质子*vs* 光子（IMRT）治疗肺癌的V20和V10数据，异质性分析*P*值分别为0.98和0.47，均采用固定效应模型分析，结果显示：V20和V10比较：*MD*=-3.70，95%CI: -5.31--2.10，*P* < 0.001；*MD*=-8.86，95%CI: -10.74--6.98，*P* < 0.001，质子明显优于光子（IMRT），差异有统计学意义；2篇文章提供质子*vs* 光子（IMRT）治疗肺癌的V5数据，采用随机效应模式分析，其结果为*MD*=-20.13，95%CI: -27.11--13.14，*P* < 0.001，质子明显优于光子（IMRT），差异有统计学意义（图 3和图 4）。

**1:**
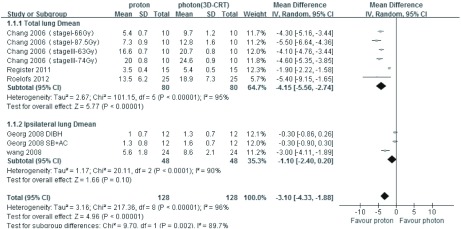
质子*vs*光子（3D-CRT）治疗肺癌双侧肺D_mean_、患侧肺D_mean_的比较 The total lung D_mean_ and ipsilateral lung D_mean_ comparing between proton beam and photon beam (3D-CRT) for lung cancer

**2:**
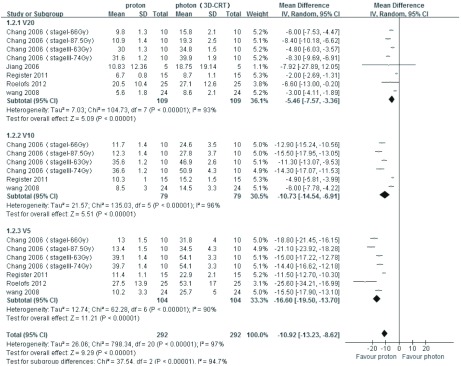
质子*vs*光子（3D-CRT）治疗肺癌V20、V10、V5的比较 The V20、V10 and V5 comparing between proton beam and photon beam (3D-CRT) for lung cancer

**3:**
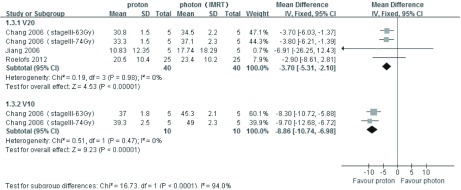
质子*vs*光子（IMRT）治疗肺癌V20、V10的比较 The V20 and V10 comparing between proton beam and photon beam (IMRT) for lung cancer

**4:**
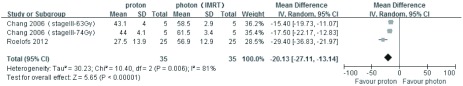
质子*vs*光子（IMRT）治疗肺癌V5的比较 The V5 comparing between proton beam and photon beam (IMRT) for lung cancer

#### 2.2.3 危及器官食管和心脏的剂量分布比较

3篇文章提供质子*vs* 光子（3D-CRT）治疗肺癌的危及器官食管D_mean_，异质性分析*P*值为0.84，采用固定效应模型分析，结果为*MD*=-2.68，95%CI: -3.50--1.85，*P* < 0.001，质子治疗食管受照射剂量明显低于光子（3D-CRT），差异有统计学意义；3篇文章提供质子*vs* 光子（3D-CRT）治疗肺癌的危及器官心脏D_mean_，异质性分析*P*值为0.005，采用随机效应模型分析，结果为：*MD*=-8.35，95%CI=-15.22--1.49，*P*=0.02，质子治疗心脏受照射剂量明显低于光子（3D-CRT），差异有统计学意义（图 5、图 6）；2篇文章提供质子*vs* 光子（IMRT）治疗肺癌的危及器官食管D_mean_，异质性分析*P*值为0.88，采用固定效应模型分析，结果为*MD*=0.08，95%CI=-5.03-5.19，*P*=0.98，差异无统计学意义；2篇文章提供质子*vs* 光子（IMRT）治疗肺癌的危及器官心脏D_mean_，异质性分析*P*值为0.93，采用固定效应模型分析，结果为*MD*=-3.94，95%CI: -6.66--1.22，*P*=0.005，质子治疗心脏受照射剂量明显低于光子（IMRT），差异有统计学意义（图 7和图 8）。

**5:**
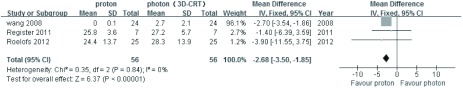
质子*vs*光子（3D-CRT）治疗肺癌的危及器官食管D_mean_的比较 The esophagus D_mean_ comparing between proton beam and photon beam (3D-CRT) for lung cancer

**6:**
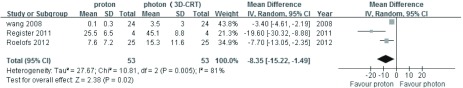
质子*vs*光子（3D-CRT）治疗肺癌的危及器官心脏D_mean_的比较 The heart D_mean_ comparing between proton beam and photon beam (3D-CRT) for lung cancer

**7:**
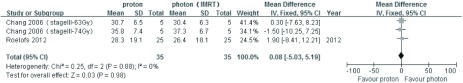
质子*vs*光子（IMRT）治疗肺癌的危及器官食管D_mean_的比较 The esophagus D_mean_ comparing between proton beam and photon beam (IMRT) for lung cancer

**8:**
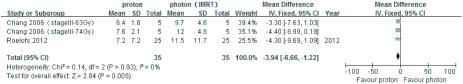
质子*vs*光子（IMRT）治疗肺癌的危及器官心脏D_mean_的比较 The heart D_mean_ comparing between proton beam and photon beam (IMRT) for lung cancer

## 3 讨论

目前肺癌放疗通常使用光子射线的3D-CRT技术和IMRT技术。本文比较了质子射线和光子射线（3D-CRT和IMRT技术）在治疗肺癌的靶区剂量分布的相关评价参数：在靶区的剂量分布上，本文纳入研究中，Wang等^[9]^的结果显示，对比质子治疗和光子3D-CRT治疗，90%的等剂量曲线均能包括99%的CTV，而对于95%的等剂量曲线，质子治疗包括了85.4%的CTV，仍有较高的靶区覆盖率，3D-CRT治疗仅包括了43.2%的CTV，说明质子治疗在一定程度上提高了射线对靶区体积的覆盖，优于光子射线3D-CRT技术，对提高靶区内剂量具有优势，这一结果与Seco等^[13]^的研究是一致的。理论上，适形指数高意味着对于靶区更高的覆盖率以及对危及器官更少的照射^[14]^，由于光子射线3D-CRT及IMRT技术使用的射束数目较质子多，可能使质子治疗对比前者在靶区适形度上有一定的不足，但本文纳入的几项研究中，在适形度的比较上，结果各不相同，Jiang等^[7]^的研究结果为，质子治疗的靶区适形度与IMRT计划相似，优于3D-CRT技术；Georg等^[10]^研究称，质子治疗靶区适形度与3D-CRT相似，均无IMRT的适形度好；而Roelofs等^[12]^研究结果则表明为3D-CRT的适形度比质子治疗优越。对于质子治疗与光子治疗在适形度上的比较，结果尚不一致，期待更多研究结论。本研究纳入的6篇文献中，仅有一篇文章描述了对靶区均匀度的比较，Jiang等^[7]^的结果为，IMRT计划比3D-CRT和质子治疗对于靶区的照射更加均匀。

对于危及器官受量的分析中，除质子治疗与光子3D-CRT治疗对比患侧肺D_mean_和质子治疗与光子IMRT治疗对比食管D_mean_这两个评价指标的差异无统计学意义外，对于双肺D_mean_、V20、V10及V5等大多数危及器官评价指标，质子治疗均有着明显优势，特别是在与3D-CRT技术的比较中，质子治疗在剂量学参数上优势更大。在临床治疗中，V20^[15]^、V10^[16]^及V5^[17]^是放射性肺炎的预测因子，如计划中降低了V20、V10及V5，对于预防和减少放射性肺炎的发生有重要的临床意义。

通过提高靶区的剂量和进一步减少危及器官的受照剂量，可以使质子治疗的剂量学优势转化成肺癌治疗预后的改善，减少放疗副反应的发生。在纳入本研究的6篇文献中，Georg等^[10]^和Register等^[12]^两项研究都进行了SBRT（体部立体定向放射治疗）与PBT的剂量学比较，结论均为质子治疗减少了危及器官的剂量。一篇发表于2010年针对治疗Ⅰ期不能手术的非小细胞肺癌进行临床观察研究的*meta*分析^[18]^在没有纳入RCT的情况下，比较了患者采用质子治疗、3D-CRT技术及SBRT在5年生存率上的差别，结论是质子治疗明显优于3D-CRT（5年生存率40% *vs* 20%），与SBRT相似（42%）。但由于该*meta*分析中纳入的均为非随机对照研究，三种照射技术的等效生物学剂量不等，其研究结论有待更多的RCT研究结论支持。一篇针对SBRT治疗肺癌的研究报道了当肿瘤受照射的生物学剂量高于100 Gy时，将获得91.9%的局控率及88.4%的3年总生存率^[19]^。Machtay等^[20]^研究发现在非小细胞肺癌的放疗中，等效生物学剂量与局控率及生存率具有明显的相关性，当等效生物学剂量提高1 Gy时，患者的局控率及生存率大约提高4%。Chang等^[21]^的一项关于Ⅲ期肺癌质子治疗同步化疗的Ⅱ期临床研究中，采用74 Gy的靶区照射剂量，化疗方案为紫杉醇及卡铂周疗，研究结果为1年生存率及无疾病进展率分别为86%和63%，同时没有发生与质子治疗相关的4级-5级不良反应。Oshiro等^[22]^对57例Ⅲ期肺癌患者进行单纯的质子治疗（剂量范围：50 Gy-84.5 Gy），有6例患者产生3级以上肺毒性，食管毒性均≤2级，没有发生心脏毒性。Hoppe等^[23]^的研究结论为接受质子治疗的Ⅲ期肺癌患者发生的毒性反应也是可耐受的，其长期生存情况还未报道。由于质子治疗对危及器官的照射剂量小，为进一步提高肿瘤的生物学剂量创造了条件，可能取得更好的临床获益。目前在MD Anderson肿瘤治疗中心正在进行一项早期非小细胞肺癌质子治疗与光子SBRT治疗的前瞻性随机对照研究^[3]^，期望获得更好的生存获益。

为了更好的提高靶区剂量，避免放射损伤，特别是在使用单次大分割模式的质子治疗中，必须要考虑到质子射线有限的高剂量区这一物理性质，以及在肺癌治疗中由于呼吸运动带来的靶区位置变化这一关键问题，已有多篇文献报道^[2, 24, 25]^，当照射靶区的密度发生改变，会造成质子射线能量的衰变，这个潜在的问题对质子治疗的精确性是一个重大的挑战。随着4D-CT的应用，获得肺癌的内大体肿瘤靶区（internal GTV, IGTV），能更好的包括肿瘤靶区^[26]^，可以在一定程度上避免肿瘤漏照问题。

本篇*meta*分析由于纳入文献的数量、数据有限，文献的级别不高，可能会对结果产生一定的偏倚，另外没有进一步对不同期别的肺癌进一步汇总分析，有望在今后纳入更多的研究，针对不同期别的肺癌分别进行统计，可能会得出更准确的结论。目前质子治疗的研究逐渐增多，在肺癌方面，放疗的各项新的技术与质子治疗相结合（例如质子调强治疗^[27, 28]^），使质子治疗的疗效更加值得期待。本文结论得出质子治疗在剂量学参数上明显优于光子，但如何体现在患者生存预后的优势上，还需要更多的临床研究及随机对照研究去验证。
